# Advancing planetary health graduate medical education assessment and evaluation methods: leveraging the entrustable professional activities model in a pediatric residency program

**DOI:** 10.3389/fpubh.2026.1804998

**Published:** 2026-04-10

**Authors:** Charles E. Moon, Sofía Curdumí Pendley, Cecilia S. Alcala, Hannah Thompson, Eleanor Bathory, Sandra Braganza

**Affiliations:** 1 Department of Environmental Medicine, Icahn School of Medicine at Mount Sinai Hospital, New York, NY, United States; 2 Department of Pediatrics, The Children’s Hospital at Montefiore, Bronx, NY, United States; 3 Department of Family and Social Medicine, Montefiore Health System, Bronx, NY, United States

**Keywords:** entrustable professional activities, environmental health, graduate medical education, One Health, planetary health

## Abstract

**Introduction:**

Planetary health content in graduate medical education (GME) is growing, mainly through incorporation of climate health content due to the evolving urgency of patient needs in response to crisis. As content expands, more objective measures of GME trainee assessment are required to evaluate trainee skill application in practice.

**Evaluation design:**

We propose a multi-stage evaluation and assessment protocol to improve objective measurement of planetary health education outcomes in a single pediatric residency program beginning in 2026. Using quality improvement methodology and an environmental health history and resource referral toolkit, we will work to improve planetary health clinical communications in pediatric well child visits. Retrospective chart review data of climate related clinical communications will then be linked with additional assessment activities using the Entrustable Professional Activities (EPAs) and Competency Based Medical Education frameworks linked to existing Accreditation Council of Graduate Medical Education Competencies and Milestones. Upon successful integration of the evaluation protocol, faculty Clinical Competency Committee meetings will have access to objective assessment data to inform assignment of EPA Supervision Levels on planetary health-informed clinical competencies.

**Discussion:**

Educating trainees on planetary health topics, linked to expertly vetted tools and resources designed for easy use in clinical encounters, will help translate education into practical clinical skills. These competencies can then be assessed using the planetary health-informed EPA framework for residency training. This protocol can serve as an example for GME innovators who wish to incorporate expanding planetary health knowledge into their curricula with more rigorous assessment grounded in evolving EPA methodology.

## 1 Introduction

Healthy environments where people can live, work, and play are essential foundations for building and maintaining health and wellbeing. Within medicine and healthcare, this concept can be referred to as environmental, climate, planetary health, or One Health. Planetary health, which has been defined as a solutions-oriented framework that aims to achieve the highest attainable standards in health and well-being worldwide, connects human health to the broader systems that sustain life (1). OneHealth similarly connects human health with animal, plant, and ecosystem health through the recognition that people are closely connected to the animals they live with and around and share the same environment that is integral for all life (2). These concepts connect human health and wellbeing to complex issues that include global warming, water, soil, food and air pollution, changing zoonotic disease epidemiology, ocean acidification, antimicrobial resistance, biodiversity loss, changes in land use and land degradation, as well as the modification of biogeochemical flows of elements like nitrogen and phosphorus on Earth (3). Consistent with the World Health Organization’s determination of global warming as the greatest threat to global health in the 21st century, most planetary health medical education has prioritized incorporating this content (4). In undergraduate medical education, U.S. MD-granting medical schools have been developing and incorporating climate health education into their curricula, with American Association of Medical Colleges (AAMC) data reporting growth from 27% of medical schools in 2019 to 69% in 2023 (5). However, similar statistics are not available for U.S. medical specialties from The Accreditation Council for Graduate Medical Education (ACGME), and reports of individual residency programs implementation of climate health curricula are growing but still limited.

Educating undergraduate and graduate medical trainees on planetary healthcare is complex and challenging. Future physicians must understand the multitude of pathways linking worsening physical and mental health outcomes to extreme heat, worsening air quality, increased frequency and severity of extreme weather events, and changes in infectious disease epidemiology. Furthermore, it requires linking this medical knowledge to relevant geographic knowledge of local climate and pollution hazards, patient-specific occupational or developmental stage-specific exposure profiles, and social determinants of health indicators to identify high-risk patients. This all must filter into more informed, specialty-specific clinical reasoning to formulate accurate differential diagnoses, strengthen shared clinical decision making, and ultimately improve the quality of acute illness treatment and chronic disease management. Additionally, physicians must understand and adapt to healthcare system disruptions from planetary health disasters to ensure quality care is still delivered. The U.S. healthcare system is composed of complex and heavily interconnected organizations and entities; resilience to these disruptions requires understanding that disruptions in one part of the system cascade among the various components and can result in unanticipated impacts in other areas. These essential planetary health professional activities require new methods of training and assessment to ensure that medical education results in physicians who are prepared for an evolving healthcare landscape.

### 1.1 Planetary health in graduate medical education

Current examples of planetary health curricula in graduate medical education (GME) span several specialties but have been primarily reported in pediatrics, family, and internal medicine programs (6–13). Most planetary health content is focused on climate health issues, linked to published frameworks, though education format and delivery methods vary between institutions and training programs (14, 15). In GME, as opposed to undergraduate medical education, there must be more variability in content and delivery format as trainees enter residency programs where knowledge and skills become increasingly specialized. Planetary health curricula should expand to use this more inclusive and solutions-focused framing centered on the health effects of environmental degradation, adapted for relevance in specific training contexts (5). Improving specialty-specific planetary health curricula creates a challenge for GME educators competing with other emerging medical knowledge for space in residency program education programming. Given the difficulty of creating a universal planetary health curriculum spanning all GME settings, educators should identify relevant content, best practices, and develop curricular standards within their disciplines. This process would be assisted by concurrent improvements in evaluation and assessment methods that center patient and trainee priorities to better narrow content and determine how essential competency outcomes are being addressed throughout training. The overarching goal is one of meeting evolving patient health needs linked to planetary health and ensuring that trainees meet these new standards without overburdening the general curriculum or current evaluation systems in training programs.

Standard assessment methods to evaluate planetary health competencies have not yet been established. Recent climate health specific GME curricular interventions have relied mainly on pre/post-survey instruments with non-standardized Likert scale questions focused on subjective knowledge, attitudes, and confidence to engage in climate clinical communications and adapt clinical practice (9–11). Likert scales have limitations as ordinal data sets and are a basic method of evaluation subject to social desirability and recall biases and more objective evaluation methods are required to better assess trainee knowledge, skill and competency growth (6, 16). The pediatric and family medicine residency programs at the University of Buffalo implemented an ongoing climate health curriculum in May 2023 with longitudinal evaluation including knowledge and attitude-based assessments, case-based exercises, reflective writing with grading rubrics, and patient encounter assessments. Initial data from this evaluation has shown variable knowledge proficiency in climate health topics, that trainees did not feel comfortable discussing climate health topics with patients, and most were graded as “not yet competent” in graded rubric evaluations (12). This is an important early example of a residency program gathering longitudinal, objective data to assess planetary health education effectiveness to inform curricular adaptation as needed based on trainee feedback to meet desired learning outcomes.

### 1.2 Advances in GME evaluation and assessment methodology

Given these challenges in early GME implementation of planetary health content, it is important to assess how medical education evaluation is evolving. The AAMC and the ACGME have embraced competency-based medical education (CBME) as the future of evaluation and assessment of trainees (17). The keystone of the CBME approach is the Entrustable Professional Activities (EPAs) framework, which puts patient needs at the center of training to guide education priorities (18). This EPA framework can be leveraged to iteratively improve planetary health competency evaluation methods, as an Association of Pediatric Program Directors (APPD) working group recently suggested; they proposed 42 climate health assessment activity examples linked to General Pediatrics EPAs and the existing ACGME Core Competencies and Milestones framework (19). This is a critical step forward in building an informed and skilled physician workforce prepared to address patient concerns linked to planetary health. The American Board of Surgery has already begun implementation of EPAs across all residency programs with plans to require EPA summative assessments to be used in initial certification decisions in 2028 (20). The American Board of Pediatrics (ABP) has also generated 12 general pediatrics EPAs still being piloted in pediatric residency programs (21). The ABP also plans to use EPA summative assessments generated by Clinical Competency Committee (CCC) meetings in initial board certification decisions in 2028 (22). This presents an opportunity to explore EPA’s utility in improving medical education outcomes in emerging planetary health topic areas.

### 1.3 Advancing planetary health GME evaluation and assessment methods

In the 2022–2023 academic year, a climate health curriculum was introduced into the pediatric and family medicine residency programs at Montefiore Medical Center in New York City. This included a pilot evaluation using pre- and post-session Likert scale surveys. Sixty-eight residents (63% survey response rate) self-reported improvements in understanding of climate health effects as well as confidence using local climate health anticipatory guidance and community resources after receiving climate health education (9). While the results were a reassuring first step, we plan to iteratively improve our climate health curriculum within the pediatrics residency program. Here, we describe our protocol to begin a new planetary health GME evaluation and assessment framework using the EPA model, with plans to begin implementation in July 2026. We hope this new framework will help advance planetary health training for pediatric trainees to meet evolving patient priorities and needs.

## 2 Pedagogical frameworks and format

CBME has 5 core components: (1) an outcomes based competency framework (historically measured by ACGME milestones and competencies, but in this project EPAs), (2) a progressive learning experience sequence building in a logical method, (3) tailored learning to individual trainee needs, (4) teaching tailored to the intended training outcome, and (5) programmatic assessment using as many data points from as many sources as possible to complete the most comprehensive picture of a trainee’s ability to perform as a physician (23). EPAs are discrete outcomes outlined as observable, routine activities that a physician should be able to perform safely and effectively to meet patient needs. Upon graduation, the goal is that newly independent physicians are able to perform these EPA activities safely and effectively without supervision (18). Using the APPD working group suggested climate health informed EPA assessment activities, we will use the CBME framework to begin evaluating planetary health learning outcomes longitudinally in future pediatric residency training yearly cohorts.

### 2.1 Overview of implementation phases and Phase 1: planetary health curriculum

**Figure 1:**
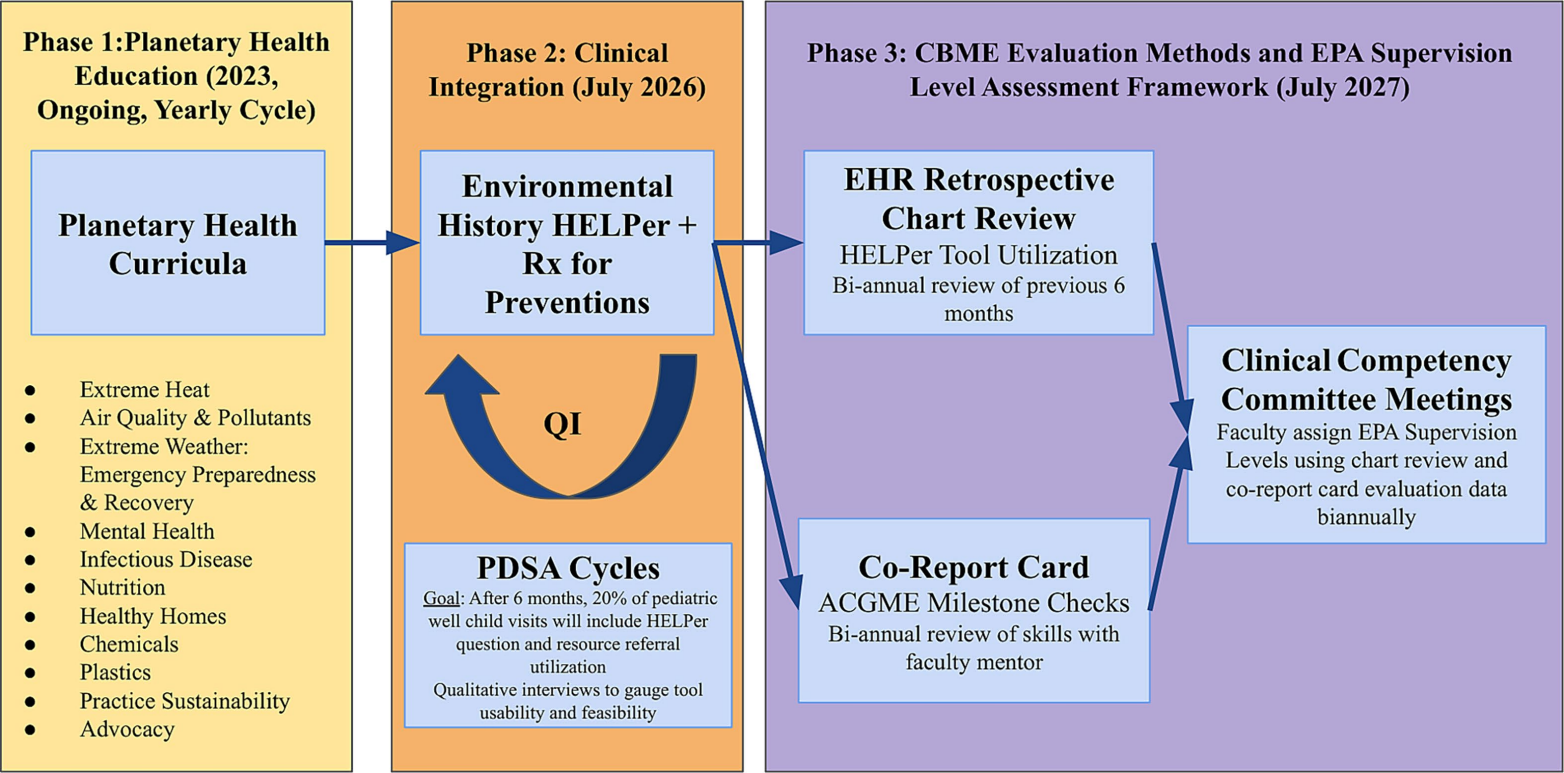
Multi-phase planetary health education evaluation and assessment implementation protocol. This protocol will test the hypothesis that a planetary health curricula, linked to translational tools with expertly vetted local resources will assist trainees advance environmentally informed clinical skillsets and prepare them for independent practice.

This evaluation and assessment framework will occur over multiple implementation phases (Figure 1). Phase 1, continuous implementation of the planetary health GME curriculum in the pediatric residency program, includes iterative expansion of planetary health content expanding from a current climate health focus. This includes ongoing incorporation of feedback from each cohort on desired content and adapting delivery formats. Phase 2 will focus on integrating environmental health history taking questions and resources into regular outpatient pediatric well childcare encounters using Plan, Do, Study, Act (PDSA) cycles. Once clinical integration of history taking and resource referrals has been implemented, Phase 3 will occur. Phase 3 will be a longitudinal evaluation using the chosen planetary health EPA assessment activities. This will begin in the 2027–2028 academic year. Phase 3 will include gathering clinical communications documentation data using retrospective chart review as well as ACGME milestone checks. This information will feed into the CCC EPA Supervision Level on the chosen planetary health EPA assessments to assign trainee competency levels.

### 2.2 Phase 2: clinical integration of an environmental health history and resource tool

One of the foundational components of providing medical care is history taking to identify relevant information to aid diagnosis, treatment, and care management. Environmental health history questions are not commonly taught or practiced in medical schools where medical history skill training is usually covered in introduction to clinical medicine courses, but are the essential foundational skill required to engage in clinical environmental health practice (24). Without baseline environmental health history taking skills, other chosen assessment activities in Table 1 on patient specific counseling (special healthcare needs, chronic conditions, local geography and exposure patterns) and connection to relevant resources are difficult to execute. With that in mind, we will introduce selected environmental health history questions in new education sessions linked to curated resources on each topic and then, using structured PDSA cycles, work with trainees to improve documentation of EH questions and resource referrals into clinical documentation during eligible visit notes. In the future, this environmental health history taking competency may be standardized within medical schools by the AAMC as an essential component of healthcare delivery, but at this time requires special focus in GME to ensure that trainees have mastered this building block to advance further skills.

**Table 1:** Selected general pediatrics entrustable professional activities and climate-informed assessment activities.

General pediatrics entrustable professional activity	AAPD working group suggested assessment activity	ACGME milestone
EPA #1 Providing preventative primary care for children of all ages	Include patient specific counseling on environmentally related exposures and threats during routine health promotion	ICS1
EPA #2 Providing comprehensive primary care for children with complex, chronic or special healthcare needs	Ensure families of children and youth with complex, chronic, or special healthcare needs have access to public health and disaster alerts	ICS1
EPA #3 Manage patients with common acute diagnoses	Inclusion of relevant environmental exposures and determinants of health in routine history taking	PC1
EPA #3 Manage patients with common acute diagnoses	Consider vulnerability of patient (age, chronic conditions, special healthcare needs, equipment and medication needs) and family (local geography, exposure patterns, pet or livestock concerns, access to adaptive resources) to climate-related shocks & stressors in care plans, including multidisciplinary teams (community health workers, home health aides, social workers, therapists, case managers, clinicians, veterinarian services as available) with community support services where necessary and available	PC5
EPA #11 Promote equitable care at the level of each individual patient and population to address racism and other contributors to health inequities	Identify and refer patients to local community resources that partner with families to address inequities in social and environmental determinants of health	SBP3

Each assessment activity was selected to fit local patient and residency program clinical care needs from the list of suggested activities proposed by the APPD Working Group (18). Each assessment activity has also been linked to existing ACGME milestones and competency based assessment frameworks.

To accomplish Phase 2, we will introduce a new clinical toolkit, the Environmental Health HELPer (Table 2). This toolkit was specifically designed for this project and consists of a series of environmentally related history-taking questions in 4 categories (Housing, Emergency Preparedness, Learning, Personal Safety categories). The HELPer tool pulls from existing pediatric environmental health history questionnaires pertinent to local priorities and are linked to expertly vetted Prescriptions for Prevention, so that clinicians can immediately respond to answers to selected history questions from previous published screening models (25–28). HELPer tool questions and linked resources will be introduced into the electronic medical record as “dot phrases,” or preformed blocks of texts that can be quickly inserted using keyboard shortcuts, allowing trainees to easily add to the history and plan sections of the clinical note and allow for retrospective chart review for objective tracking of tool use. Full questionnaire usage is not expected, users will be directed to ask any question from the HELPer tool during a well child visit (not every visit) and delivery the resources linked to question responses. To gauge feasibility, the frequency of specific question usage will be tracked to see which are most commonly used to determine relevancy and redesign of infrequently used questions. Education sessions will introduce the tool components to trainees and structured PDSA cycles combined with semi-structured interviews conducted before and 6 months after HELPer tool introduction will allow identification of uptake barriers that can be sequentially addressed.

**Table 2:** Environmental health history HELPer dot phrase tool.

Environmental history HELPer	
History component *document answer next to questions used*	Housing How do you control the temperature in your home when it gets too hot or too cold? Are they ever too expensive to use? Do you use a gas stove when cooking for your family? Do you have a range hood you can turn on while cooking? Can you describe your cleaning practices in your home? Are there any environmental issues in your home that concern you? (smoke, mold, pests, water damage)? Emergency preparedness and environment Do you have environmental concerns about your child’s school? How much time does your child spend outdoors for work, sports, school, or for fun? If there is a power outage, do you have a plan for refrigerated medications and/or electronic medical devices? Do you have a disaster plan in case of extreme weather and you need to leave your home? Do you have an evacuation plan for your family? Your pets? Learning Where do you get information about how to protect your health from extreme weather (heat waves, poor air quality, flash flooding)? Do you know if your home is in a flood or hurricane evacuation zone? Do you know where your local cooling or evacuation center is? Personal safety Do you have pets in your home? Are they properly vaccinated and receive regular tick control treatments? Is there a source of pollution that worries you in your community? Do you have a smoke alarm, carbon monoxide alarm, and natural gas detector in your housing unit? Do you have someone who can check on your family during an emergency?
Plan component	Given the relevant history obtained using the Environmental History HELPer, I recommended the following resources to the family/patient: [drop down list of Resources] I had time to discuss these resources with the family AND/OR Given time restraints of the visit I asked family to review the resource and contact me with any questions/bring up for discussion at their next visit AND/OR referred to PEHSU for further expert consultation.

Questions are organized in Housing, Emergency Preparedness and Environment, Learning, and Personal Safety sections. The history dot phase section can be introduced into the social history portion of the patient’s note. The user can delete unused questions or write responses behind questions they asked during the visit. The plan section can be linked to social determinants of health relevant clinical codes in the problem list and allows documentation of specific resources provided and level of discussion or a referral is made. Resources from drop down list found in Supplementary Table 1.

### 2.3 Phase 3: implementing CBME and EPA based planetary health assessment framework

After determining the best local methods to clinically integrate environmental health history taking and resource referrals during Phase 2 activities, we will begin Phase 3. Phase 3 will link this retrospective chart review system into the broader planetary health CBME evaluation framework through EPA assessments. To start, we have selected 4 General Pediatrics EPAs linked to 5 climate-informed assessment activities, which are also linked to 4 ACGME Competencies and Milestones (Table 1). We chose assessment activities for each EPA based on the following: (1) Assessment activities are feasible to observe and measure within general pediatrics training during residency in regular well child outpatient care visits (2) assessment activities are foundational components of engaging in environmental health clinical care and/or (3) assessment activities allow for direct observation of skill use through faculty precepting of patient visits.

**Figure 2:**
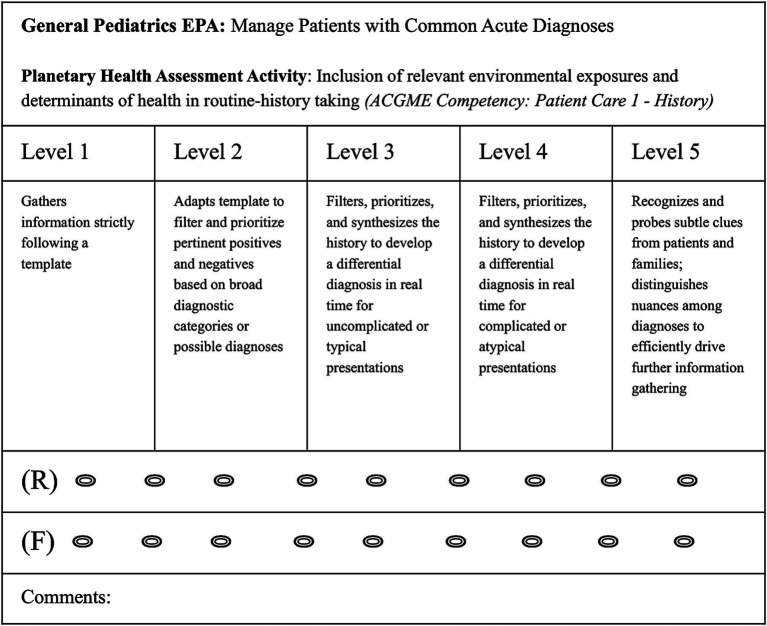
Individualized Learning Plan (ILP) Co-Report Card Example. In biannual ILP meetings, faculty (F) and resident trainees (R) will fill out ACGME Milestone scales connected to the planetary health assessment activities and relevant General Pediatrics EPA. During ILP meetings, this can be used to scaffold mentor/mentee conversations about differences in competency levels and how the dyad can work together to help advance the trainees skills.

**Figure 3:**
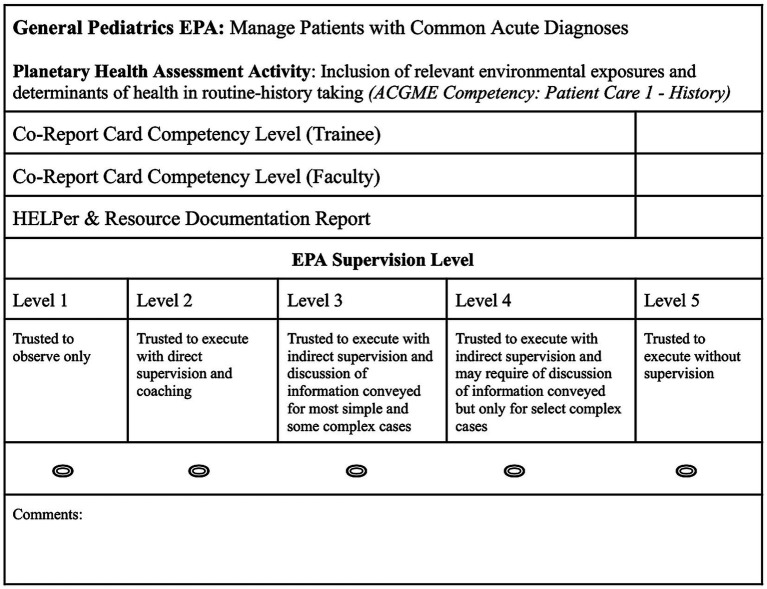
Clinical Competency Committee Entrustable Professional Activities Supervision Level Card Example. For each selected General Pediatrics EPA and associated climate-informed assessment activity, Co-Report Card grades from ILP meetings with both faculty and trainee scores will complement HELPer question and resource referral documentation from retrospective chart review over the previous 6 month training period, allowing CCC members to better assess overall trainee competency as assigned through a specific EPA Supervision Level.

In addition, during individual learning plan (ILP) meetings between trainees and faculty that occur biannually, both trainee and faculty mentor will complete a short, 5 question “Co-Report Card” on each EPA climate-informed assessment activity linked to specific ACGME Milestones and Competencies (Figure 2). This process will allow residents to self-assess their strengths and weaknesses, tailor professional goals, and co-develop individual plans to achieve competency with their faculty mentor. Trainees enter GME with different strengths and growth opportunities, and this co-generation of feedback allows both mentor and mentee to reflect on the mentee’s progress and collaborate on how to refine their learning plan in these areas as needed (29). Together, ILP Co-Report Cards and retrospective chart review data since the last CCC meeting will be triangulated and provided to CCC members who will, in addition to this objective data gathered, use clinical observation to assign an EPA Supervision Level in each assessment area (Figure 3). This can be tracked twice yearly over the course of residency education to see how trainee competency evolves over time and help identify trainees who need additional education or support to meet their learning goals.

### 2.4 Learning environment

This new framework will be implemented within the Social Pediatrics Residency Program at The Children’s Hospital at Montefiore in Bronx, New York. This is an ACGME-accredited residency program of 12 residents that is integrated into a larger categorical program with 62 additional pediatric residents at Montefiore over 3 years of GME training. Social Pediatrics residents have additional primary care clinical time at the Comprehensive Health Care Center, located in the South Bronx, which serves a population with high social determinants of health needs and are among the most vulnerable communities to climate health impacts in New York City (30, 31). It also has additional weekly protected education time outside of the regular categorical pediatrics curriculum to focus on social medicine topics that impact patient health. These characteristics make the Social Pediatrics Residency Program well positioned to test this evaluation framework that can be later scaled to the larger categorical program.

Social Pediatrics residents currently receive climate health education in the categorical pediatrics education programming (two 45-min lectures twice yearly). In addition to this, further planetary health and advocacy content will be included in their additional educational time (4–5 60 min lectures annually) and short (10–15 min) clinic lectures focused on the HELPer tool clinical integration. This curriculum is iteratively redesigned annually to reflect feedback from residents and faculty, new planetary health medical literature, and changes in community referral resources. Regardless of content change, the curriculum continues to use a preventative medicine lens that is focused on outpatient care.

Residency program faculty will also receive education sessions on the HELPer tool questions and resources during Phase 2. They will receive development sessions prior to Phase 3 to familiarize themselves with the new EPA framework, EPA Supervision levels, and the chosen data collection methods (retrospective chart review of HELPer tool use and bi-annual ILP Co-Report cards) that will then feed into the CCC meetings where EPA Supervision level determinations are made. This is an opportunity to prepare faculty for new EPA standards and methods of assessment that will be used in residency program evaluations across all subject and competency areas, while serving as continuing medical education for faculty who have not received planetary health education in their medical training. Through the process of observing and assessing trainees on these specific EPAs, we believe that faculty will gain planetary health practice knowledge and skills as commonly referred to by the “see one, do one, teach one” teaching model. We plan to test this hypothesis with faculty over the course of Phase 3 implementation, but further description is beyond this article’s scope.

## 3 Results to date and plan for further data collection

The planetary health curriculum in the Montefiore Pediatrics and Social Pediatrics residency programs was initiated by the first author in 2023 with detailed curriculum structure, content, learning objectives, and initial evaluation data published in December 2024 (9). The HELPer environmental health history taking toolkit has been developed and consists of 12 questions linked to existing Prescription for Prevention and other community resources (Table 2, see Supplementary material for HELPer questions linked to identified community resources). Currently, we plan to implement the HELPer tool quality improvement project into pediatric well child visits in July 2026. We will conduct monthly structured PDSA cycles that begin with short education sessions on 1–2 HELPer questions and associated resources, with HELPer documentation in pediatric well child visits reviewed monthly. The goal is for pediatric trainees to document any HELPer question usage and linked resource referrals in at least 20% of pediatric well child visits by the end of the initial 6-month quality improvement period. This is based on the assumption that trainees are not currently asking and documenting environmental health history questions in the electronic health record and evidence suggests that initial quality improvement metrics can only be expected to improve by 20% during initial cycles (32).

We will track HELPer usage through retrospective chart review of eligible visits and collect de-identified data on patient age, gender, insurance type, and race/ethnicity to observe possible differences in HELPer tool use among various demographics. We will assess the usability of the HELPer tool and gauge its effectiveness during PDSA cycles by speaking with trainees and faculty and using a standardized checklist compiled from the semi-structured interview guide. Changes that occur from PDSA cycle feedback may include changes in question word choice, dotphrase formatting, how the tool is documented in the electronic health record, changes in resource formatting or changes to tool education content and methods. Pre- and post-semi-structured qualitative interviews will be conducted prior to and 6 months after HELPer tool introduction to explore tool acceptability and usability attitudes (semi-structured interview guide included in Supplementary material). Interview transcripts will be recorded and analyzed using an inductive grounded theory approach that identifies themes using a constant comparative method to examine patterns as well as areas of convergence and divergence. Trainee confidence in translating environmental health knowledge into practice and providing environmental health resources will be assessed in the semi-structured interviews to help determine tool effectiveness.

We will define successful HELPer toolkit implementation within pediatric well child visits when we reach an average tool usage rate of at least 20% by trainees over a 3-month period. Using this goal and feedback received from the PDSA cycles and semi-structured interviews, we will determine that trainees become comfortable engaging in environmental health communications before formally incorporating this data into initial planetary health EPA supervision level decision-making during CCC meetings. This will be supplemented by the faculty and trainee biannual Co-Report Card data. This assessment system is anticipated to begin implementation in the 2027–2028 academic year, and we aim to collect EPA supervision levels over multiple resident cohorts through at least 2030. This data will help gauge the effectiveness of the planetary health curriculum and inform further iterative changes in the curriculum and toolkit.

## 4 Discussion

This protocol to improve trainee assessment and evaluation of planetary health clinical skills is an important step forward in advancing planetary health knowledge into clinical practice. It can serve as an example for other GME leaders who wish to improve their assessment of trainee skills relevant to planetary, climate, and environmental health-themed training outcomes. Skills measurement combined with adaptive teaching methods that are responsive to changing patient and community priorities are likely to lead to better trained physicians, improve care delivery, and contribute to better patient health outcomes. While this example is focused in a pediatric training setting, the CBME and EPA frameworks can be adapted into any GME discipline.

The EPA framework helps advance CBME through focusing on the needs of patients and putting those priorities at the center of education and training. As medical knowledge continues to expand, the EPA framework allows for quick identification of educational gaps and quality improvement methods to address them. While we focus on 5 specific assessment activities linked to 4 EPAs and 4 ACGME milestones in our initial rollout of this new learner assessment protocol, the APPD working group suggested 42 different assessment activities, and more assessment activities can likely be identified (19). Given the breadth of clinical information covered in packed GME curricular standards that physicians are required to know, as well as other evolving patient needs that also require improved skill assessments, it is challenging for any residency program to fully evaluate and assess all EPAs. However, specific assessment activities can be identified and prioritized at different times for increased evaluation based on patient and community priorities. Once an educational intervention targeting a specific EPA assessment activity has regularly demonstrated that learners reach the required skill level for independent practice, it can be removed from the evaluation cycle and other evaluation priorities can be inserted in its place. Conversely, if learners are not reaching required competencies during training, those become opportunities to improve the curriculum or develop more specialized learning plans with trainees.

Initial General Pediatrics EPA implementation in 23 residency programs among 1,987 pediatric trainees showed that for roughly half of the EPAs (which decreased from a total of 17 to 12 in a 2nd iteration published by the ABP in 2025), at least 89% of were rated as able to practice 9 EPAs unsupervised by time of graduation (33). However, at least 30% of graduating residents did not meet the level of unsupervised practice in the EPAs for providing consultation, transition to adult care, behavioral and mental health, as well as practice management (33). The authors identified these areas as known gaps in pediatric training and care and suggested that trainee curriculum in these areas required significant improvement. We expect this reasoning to also hold true for planetary health EPA assessments in the future. GME planetary health curricula are new content being introduced to learners, and the evidence base for planetary health impacts continues to rapidly expand. For planetary health EPA assessment activities that are not met by time of graduation, educators may need to adapt their curricula to better address patient and learner needs. Another option is to embrace the philosophy of life-long learning and plan for additional training opportunities and evaluation in fellowship training and within continuing medical education (CME) avenues. The EPA framework allows for learners to embrace a growth mindset and gradually obtain mastery in more complex skillsets (34).

There is a need to gather more robust data in medical education settings that better inform EPA supervision level assignments and gauge readiness for independent practice. Residency programs will be required to generate, track, gather, and organize large amounts of data for CCCs on the General Pediatric EPAs so faculty can make the most informed entrustment decisions. Depending on the methods of assessment used (such as knowledge tests, role plays, observed standardized clinical encounters, enhanced direct observation and feedback, chart review, and/or reflective essays) this may generate more data points that require further data management to input into EPA-level decision making. In our own example, 12 Social Pediatrics residents may see between 30 and 40 well child visits a month over 1–3 clinical sessions/week, requiring roughly 3,000 distinct visit notes that require review and monthly reports generated for each resident. This will add to an already high burden of assessments already done within medical education. However, there is an opportunity to incorporate emerging methods like artificial intelligence, clinical informatics, and data management practices into medical education to better address these logistical concerns and ease the assessment burden on educators and trainees alike. Currently the ABP is spearheading a large EPA pilot project with a phone-based application that allows trainees and faculty to initiate 5 question daily assessment forms on specific assessment activities and competencies, with encouraging data reported so far on feasibility and reduction in evaluation burdens, with trainees reporting more useful feedback as well (35). Further advancements in artificial intelligence dictation and clinical observation technology may also support educators and reduce evaluation burdens as they are incorporated into clinical practice settings.

### 4.1 Limitations

This assessment and evaluation protocol is planned in a small cohort in a primary care and social medicine focused residency program that has additional protected education time, which may limit generalization of this assessment protocol into larger residency programs. However, we hope to broaden this project into the larger categorical residency program at CHAM in the future and share lessons learned from broadening implementation. There may be further expansion opportunities nationally across pediatric residency programs as General Pediatrics EPA implementation moves toward the ABP 2028 mandate and beyond. This curriculum, evaluation, and assessment plan focuses on preventative medicine and primary care assessment activities, however further activities and models will need to be developed in diverse care settings including hospital medicine, the newborn nursery, and the emergency department to ensure general competency similar to other EPAs. Our assessment model only uses 2 evaluation methods (retrospective chart review and ILP co-report cards) to assist CCC members in creating summative EPA assessment decisions. Other evaluation methods may be more useful or easily implemented in specific contexts and may become part of our future assessment plan depending on trainee feedback and outcomes.

Using the EPA framework to iteratively improve planetary health education evaluation and assessment are limited by the novelty of the specific content and the evaluation framework in many residency programs who are unfamiliar with either, let alone in combination. However, the ABP mandate presents an opportunity to use this research as an introduction for faculty into the broader work of General Pediatrics EPA implementation. This could be presented as faculty development to gain support from residency programs and GME institutional leadership to implement a new system. This pilot data can also be reported back to the ACGME and ABP to help inform further EPA assessment activity development in this and other topic areas. Another challenge will be the longitudinal nature of the EPA assessment implementation phase. A multi-year medical education research protocol on new content, using new methods and tools will require sustained engagement as well as identifying essential faculty champions to assist with implementation and ensuring quality feedback is elicited. Regardless of the difficulty, medical education methods require improvement and a better focus on patient needs of physicians to deliver high quality healthcare benefits to all stakeholders. While these more objective measures may prove to be a greater administrative burden and cost in the short term, they are less subject to bias and more likely to measure outcomes that are relevant to patient care. Further research in this area will focus on whether the planetary health curriculum and EPA assessment framework leads to improved patient health literacy, positive behavioral change, and better health outcomes.

Resident trainees may also object to more frequent collection of data in chart review and Co-Report Card formats on climate health EPAs. Regardless of resident opinion, the ABP plans to implement EPAs into residency programs in the near future, and new evaluation methods requiring this level of observation and tracking will be required in other foundational competency areas. Current examples in surgery and pediatric pilots indicate that improved and more immediate feedback, as well as decreased evaluation burden for both trainees and faculty, have been instrumental for uptake (20, 33, 35). Proper introduction of the CBME and EPA frameworks to trainees, as well as why they are being implemented, will be essential prerequisites to obtain trainee buy-in to any system that introduces more tracking and observation of their growing skillset. We will also emphasize that these assessment activities and determinations will not impact trainee’s ability to graduate or become eligible for ABP board certification at this time. GME accreditation bodies will need to incorporate these essential planetary health competencies into future practice requirements to establish high quality training standards that continue to meet evolving patient needs.

## 5 Conclusion

New evaluation and assessment methods are needed to better assess medical trainees’ competencies in providing planetary healthcare and meeting evolving patient needs and priorities. This research, evaluation, and assessment protocol is one example of how graduate medical educators can iteratively improve their planetary health themed curricula through more objective data measurement tied to CBME advancements through the EPA outcome-based competency framework and be adapted as needed for specific clinical and program contexts. Future work will focus on assessing if improvements in pediatrician planetary health clinical skills as measured through the EPA framework result in improved patient health outcomes using biomarkers or other disease measures.

## Data Availability

The original contributions presented in the study are included in the article/Supplementary material, further inquiries can be directed to the corresponding author.
