# Supplementary material for: Advancing planetary health graduate medical education assessment and evaluation methods: leveraging the entrustable professional activities model in a pediatric residency program
Source: Front Public Health. 2026 Apr 10;14:1804998. doi: 10.3389/fpubh.2026.1804998 (PMC13108479; doi:10.3389/fpubh.2026.1804998)
Supplement: Supplementary file 1 [file Table_1.docx]

| **HELPer Question** | **Linked Prescription (Rx) for Prevention, Guidance and/or Other Resources** |
| --- | --- |
| How do you control the temperature in your home when it gets too hot or too cold? Are they ever too expensive to use? | If poor ventilation/indoor air quality:  [Indoor Air Quality](https://nyscheck.org/RXP/NYS/rx_english_IndoorAir_NYS_WEB.pdf) Rx  [DIY Box Fan Air Purifier Guide](https://airpurifiersadvisor.com/how-to-make-an-air-purifier-with-a-box-fan/)    Heating Issues:  [Tenant Rights](https://nyscheck.org/RXP/NYS/rx_english_TenantRightsHeat_NYS_WEB.pdf) Rx  [Space Heater Safety](https://nyscheck.org/RXP/NYS/rx_english_SpaceHeaters_NYS_WEB.pdf) Rx  [Cold Weather Safety](https://nyscheck.org/RXP/NYS/rx_english_ColdWeatherSafety_NYS_WEB.pdf) Rx    Cooling Issues:  [Heat Safety for babies](https://nyscheck.org/RXP/NYS/rx_english_extremeheatbabies_NYS_WEB.pdf) Rx  [Heat Safety for children](https://nyscheck.org/RXP/NYS/rx_english_extremeheatchildren_NYS_WEB.pdf) Rx  [Heat Safety during pregnancy](https://nyscheck.org/RXP/NYS/rx_english_extremeheatpregnancy_NYS_WEB.pdf) Rx |
| Do you use a gas stove when cooking for your family? Do you have a range hood you can turn on while cooking? | [Indoor Air Quality](https://nyscheck.org/RXP/NYS/rx_english_IndoorAir_NYS_WEB.pdf) Rx  [Gas Stoves](https://nyscheck.org/RXP/NYC/rx_english_GasStoves_NYC_WEB.pdf) Rx |
| Can you describe your cleaning practices in your home? | [Green Cleaning](https://nyscheck.org/RXP/NYS/rx_english_GreenCleaning_NYS_WEB.pdf) Rx  [Controlling Allergens in Your Child’s Bedroom](https://nyscheck.org/RXP/NYS/rx_english_Allergensbedroom_NYS_WEB.pdf) Rx |
| Are there any environmental issues in your home that concern you? (smoke, mold, pests, water damage)? | [Asbestos](https://nyscheck.org/RXP/NYS/rx_english_Asbestos_NYS_PDF_WEB.pdf) Rx  [Lead Pain](https://nyscheck.org/RXP/NYS/rx_english_Lead_NYS_WEB.pdf)t Rx  [Indoor Air Quality](https://nyscheck.org/RXP/NYS/rx_english_IndoorAir_NYS_WEB.pdf) Rx  [Mold](https://nyscheck.org/RXP/NYC/rx_english_Mold_NYC_WEB.pdf) Rx  [Safer Pest Control](https://nyscheck.org/RXP/NYC/rx_english_PestControl_NYC_WEB.pdf) Rx  [Cockroaches](https://nyscheck.org/RXP/NYS/rx_english_Cockroaches_NYS_WEB.pdf) Rx  [Dust Mite](https://nyscheck.org/RXP/NYS/rx_english_DustMites_NYS_WEB.pdf) Rx  [Controlling Allergens in Your Child’s Bedroom](https://nyscheck.org/RXP/NYS/rx_english_Allergensbedroom_NYS_WEB.pdf) Rx |
| Do you have environmental concerns about your child’s school? | [Indoor Air Quality](https://nyscheck.org/RXP/NYS/rx_english_IndoorAir_NYS_WEB.pdf) Rx  [Mold](https://nyscheck.org/RXP/NYS/rx_english_Mold_NYS_WEB.pdf) Rx  [Asbestos](https://nyscheck.org/RXP/NYS/rx_english_Asbestos_NYS_PDF_WEB.pdf) Rx  [Vaping & E-Cigarettes](https://nyscheck.org/RXP/NYS/rx_english_Vaping_NYS_WEB.pdf) Rx  [Smoking](https://nyscheck.org/RXP/NYS/rx_english_Smoking_NYS_WEB.pdf) Rx  [Marijuana](https://nyscheck.org/RXP/NYS/rx_english_Marijuana_NYS_WEB.pdf) Rx    Refer to PEHSU for specific concerns that require follow up |
| How much time does your child spend outdoors for work, sports, school, or for fun? | If child is spending significant amounts of time outdoors on hot days:    [Heat Safety for children](https://nyscheck.org/RXP/NYS/rx_english_extremeheatchildren_NYS_WEB.pdf) Rx  [Heat Safety for Athletes](https://nyscheck.org/RXP/NYS/rx_english_extremeheatathletes_NYS_WEB.pdf) Rx  [Nature and Play](https://nyscheck.org/RXP/NYS/rx_english_NaturePlay_NYS_WEB.pdf) Rx  [Insect Repellents](https://nyscheck.org/RXP/NYS/rx_english_InsectRepellent_NYS_WEB.pdf) Rx  [Lyme Disease and Tick Borne Illness](https://nyscheck.org/RXP/NYS/rx_english_LymeDisease_NYS_WEB.pdf) Rx  [Teen Tanning](https://nyscheck.org/RXP/NYS/rx_english_TeenTanning_NYS_WEB.pdf) Rx  [Sun Safety](https://nyscheck.org/RXP/NYS/rx_english_Sun_NYS_WEB.pdf) Rx  [Fordham Tri-State Tick Risk \| Fordham](https://www.fordham.edu/about/campuses/the-louis-calder-center/research/indices/fordham-tri-state-tick-risk/)    Adolescent Resources:  [Teen Workers](https://nyscheck.org/RXP/NYS/rx_english_TeenWork_NYS_WEB.pdf) Rx  [Environmental Exposures at Home & Work](https://nyscheck.org/RXP/NYS/rx_english_JobHobby_NYS_WEB.pdf) Rx |
| If there is a power outage, do you have a plan for refrigerated medications and/or electric medical devices? | Provide anticipatory guidance to families - neighbor, family, friends that still have power.  [NYC Evacuation Centers](https://data.cityofnewyork.us/Public-Safety/Hurricane-Evacuation-Centers-Map-/ayer-cga7)  [Prepare an Emergency Kit & To Go Bag](https://plannownyc.cityofnewyork.us/have-a-go-bag/) |
| Do you have a disaster plan in case of extreme weather and you need to leave your home? | If no, don’t have somewhere to go:  [NYC Hurricane Evacuation Centers](https://data.cityofnewyork.us/Public-Safety/Hurricane-Evacuation-Centers-Map-/ayer-cga7)  [NYCHA Evacuation Information](https://www.nyc.gov/site/nycha/residents/evacuations.page) |
| Where do you get information about how to protect your health from extreme weather (heat waves, poor air quality, flash flooding)? | Choose a daily weather app or website to monitor  Heat/Flooding/AQI Info: [NOTIFYNYC](https://a858-nycnotify.nyc.gov/notifynyc/)  AQI Info: [AirNow.Gov](http://airnow.gov/) or App  Flash flooding: [FloodNet NYC](https://dataviz.floodnet.nyc/) |
| Do you know if your home is in a flood or hurricane evacuation zone? | [NOTIFYNYC](https://a858-nycnotify.nyc.gov/notifynyc/)  [NYC Flood Zones \| Flood Risk \| FloodHelpNY.org](https://floodhelpny.org/)  [NYC Hurricane Evacuation Centers](https://data.cityofnewyork.us/Public-Safety/Hurricane-Evacuation-Centers-Map-/ayer-cga7)  [Prepare an Emergency Kit & To Go Ba](https://plannownyc.cityofnewyork.us/have-a-go-bag/)g |
| Do you know where your local cooling or evacuation center is? | If yes, provide resource regarding NYC [Cooling Center Network](https://finder.nyc.gov/coolingcenters/)  [NYC Hurricane Evacuation Centers](https://data.cityofnewyork.us/Public-Safety/Hurricane-Evacuation-Centers-Map-/ayer-cga7) |
| Is there a source of pollution that worries you in your community? | [Outdoor Air Quality](https://nyscheck.org/RXP/NYS/rx_english_OutdoorAir_NYS_WEB.pdf) Rx  [Indoor Air Quality](https://nyscheck.org/RXP/NYS/rx_english_IndoorAir_NYS_WEB.pdf) Rx  [Noise](https://nyscheck.org/RXP/NYS/rx_english_Noise_NYS_WEB.pdf) Rx  [PFAS](https://nyscheck.org/RXP/NYS/rx_english_PFAS_NYS_WEB.pdf) Rx  [Personal Care Products](https://nyscheck.org/RXP/NYS/rx_english_PersonalCareInfant_NYS_WEB.pdf) Rx  [Chemical Exposure at Home and Work](https://nyscheck.org/RXP/NYS/rx_english_EnvironmentalExposures_NYS_WEB.pdf) Rx  [Green Cleaning](https://nyscheck.org/RXP/NYS/rx_english_GreenCleaning_NYS_WEB.pdf) Rx    Refer to PEHSU if needed for further consultation |
| Do you have a smoke alarm, carbon monoxide alarm, and natural gas detector in your housing unit? | [Carbon Monoxide](https://nyscheck.org/RXP/NYC/rx_english_Carbon%20monoxide_NYC_WEB.pdf) Rx  Gas Stove Rx |
| Do you have someone who can check on your family during an emergency? | Counseling patients and/or family to identify people or community organizations who can check in during emergency scenarios. |

**Supplementary Table 1. Breakdown of HELPer Toolkit.** Table organized by each HELPer question linked to selected resources and anticipatory guidance for patients who are found to have relevant concerns identified during history taking. The Prescription for Prevention resources are evidence-based and expertly vetted (27).

**HELPer Tool Semi-Structured Interview Guide**

**Section 1: Explanation of Semi-Structured Interview Guide**

Thank you for agreeing to participate in this semi-structured interview. You have been invited to participate because you have expertise as a pediatrician and practical experience incorporating environmental health history questions and resources into clinical encounters. Your insights will help us implement the HELPer toolkit in clinical practice in ways that are beneficial and impactful for both clinicians and patients.

The goals of this study are to (1) provide a deeper understanding of current environmental health communication practices between pediatricians and their patients; (2) determine the extent to which the HELPer tool may support and improve upon these communications; and (3) identify best practices for integrating environmental health history taking, counseling, and resource referrals into pediatric well child visits.

We are conducting two rounds of semi-structured interviews, one prior to HELPer tool introduction in clinical encounters and another round 6 months after HELPer tool is in place. The first round of interviews will focus on (1) perspectives on environmental exposures and pediatric health outcomes, (2) current practice around discussing environmental health concerns with patient families, and (3) potential barriers or challenges related to implementing the current iteration of the HELPer tool. The 2^nd^ round of semi-structured interviews will address usability measures of the tool (including acceptability, feasibility, appropriateness, and sustainability of tool format).

We do not expect either of the 2 interviews to take longer than 60 minutes of your time (2 hours total over 6 months). We appreciate your honesty and your thoughts. Please consider this to be more like a conversation with a focus on your experience, your opinions, and what you think or feel about the topics covered. This program is voluntary, so if you at any point feel as if you would like to stop participating, you can stop at any time.

We will now review the consent form. We would like your permission to audio record this discussion. You may choose to decline to answer any question you wish.

**Section 2: HELPer Tool Semi-Structured Interviews Round 1 (Pre-tool introduction)**

We would like to start by asking:

1. When you are with your patients, what are the environmental related health issues that you see? (e.g., asthma, mold, lead, lyme disease, heat related issues, chemical exposures?)
   1. Probe: When you see an environmental related health issue, what resources or information do you provide to families?
2. Have your patients or their families independently brought up environmental health concerns with you?
   1. Probe: What environmental health issue do you think is a priority for the families that you see?
   2. Probe: In what ways do you believe your patients and families are aware that the environment can affect their health?
   3. Optional - Probe: What about your patients with asthma? Those patients who have electricity-dependent medical equipment? Patients with families who don’t have or can’t afford to use air conditioning?

Now, we would like to ask about how you stay current on emerging trends and best practices related to environmental health issues.

1. How do you receive information about emerging environmental health trends? (e.g. professional association newsletters or on-line offerings (like American Academy of Pediatrics); institutional communications or grand rounds; New York State Department of Health or other government health communications)
   1. Probe: What environmental health issue do you think is a priority for the families that you see?
   2. Optional Probe: Where and how often do you seek out information related to environmental health?
   3. Optional Probe: Do you find the environmental health lectures you’ve received help you stay current with environmental health trends in practice?
2. How have you taken environmental health information and applied it in your practice?
3. In your opinion, what are the best modes for effectively integrating environmental health history taking questions on emerging health issues into clinical care?
   1. Probe: What are suggested ways to support you and other pediatric clinicians changing your practice without adding to your administrative burden?
   2. Optional Probe: What methods/modes to integrate environmental health screening and counseling are you currently using in your clinical care?
4. The HELPer tool is designed to highlight 4 categories of environmental health questions on housing, emergency preparedness, learning (about environmental hazards), and personal safety. These questions were selected as those most pertinent to aspects of daily pediatric practice and are linked to expertly vetted anticipatory guidance and local resources that patients can be referred to. This tool is designed to be easily used through dotphrases that can be entered into the history and plan portions of clinical documentation in the electronic health record. In your opinion, do you think that this format will be helpful to reach the goal of improving environmental health communications in your practice?
   1. Probe: What methods, modes, or formats do you think would work better for asking environmental health questions in clinical settings?
   2. Optional Probe: Do you have other suggestions for ways to provide resource referrals to patients in or outside of clinical encounters?

**Section 3: HELPer Tool Semi-Structured Interview Round 2 Post-Introduction (6 months after tool implementation)**

Thank you for your participation in the Environmental Health History HELPer tool implementation pilot and for returning for another semi-structured interview to discuss your thoughts. We will start by asking you a few questions about the usability of the HELPer tool questions and linked Prescription for Prevention Resources.

1. Generally, how would you describe your experience using the HELPer tool questions and resources over the last 6 months?
2. Do you find the question content and delivery format acceptable for your practice?
   1. Probe: Are the questions too complex for easy usage? Do you feel comfortable asking these questions during clinical encounters? Why or why not?
3. In practice, how feasible have you found it to adopt HELPer tool questions and resources in pediatric well child visits?
   1. Optional Probe: Do you have specific examples of difficulties? Specific examples of how it worked well?
4. Do you find the HELPer toolkit relevant or useful for your practice during pediatric well child visits?
   1. Optional Probe: Can you provide specific examples?
5. How easy or difficult do you feel it will be to continue to use HELPer toolkit questions and resources in your future practice?
6. Are there challenges or barriers you’ve identified to using the HELPer toolkit questions and resources during well child visits?
   1. Probe: Is their clinical guidance or resources you were unable to offer during a visit because of extra time spent on HELPer tool questions or resources?
7. In your opinion, what are the best modes for effectively integrating environmental health history taking questions on emerging health issues into clinical care?
   1. Probe: Has this changed over the last 6 months since the HELPer tool was introduced?
8. What is your sense of patient’s response to these questions?
   1. Optional Probe: Are they confused why you are asking them? Appreciative? Other responses you’ve seen?

**Supplemental Material. HELPer Tool Semi-Structured Interview Guide.** Section 1 introduces the goals and expectations of the semi-structured interviews to study participants and includes obtaining consent (consent form not included). Section 2 features the first round of semi-structured interview questions prior to HELPer tool introduction. Section 3 features the second round of semi-structured interview questions that will take place 6 months after the HELPer tool has been introduced clinically.
